# Nontargeted Identification of the Phenolic and Other Compounds of *Saraca asoca* by High Performance Liquid Chromatography-Positive Electrospray Ionization and Quadrupole Time-of-Flight Mass Spectrometry

**DOI:** 10.1155/2013/293935

**Published:** 2013-08-05

**Authors:** Ashwani Mittal, Preeti Kadyan, Anjum Gahlaut, Rajesh Dabur

**Affiliations:** ^1^Department of Biochemistry, University College, Kurukshetra University, Kurukshetra, Haryana 136119, India; ^2^Department of Biochemistry, Maharshi Dayanand University, Rohtak, Haryana 124001, India; ^3^National Research Institute of Basic Ayurvedic Sciences, Central Council for Research in Ayurvedic Sciences, Nehru Garden, Kothrud, Pune, Maharashtra 411038, India

## Abstract

High performance liquid chromatography coupled with quadrupole time-of-flight mass spectrometer was used for separation and identification of phenolic and other compounds in the water extracts of *Saraca asoca* (Roxb.), De. Wilde. The aim of the study was to identify and evaluate the distribution of phenolic compounds in the different parts of the plant. The identity of compounds was established through the comparison with standards and characteristic base peaks as well as other daughter ions. In crude extracts, 34 catechin derivatives, 34 flavonoids, and 17 other compounds were identified. Interestingly, further analysis of compounds showed plant part specific unique pattern of metabolites; that is, regenerated bark is observed to be the best source for catechin/catechin derivative while flowers were found to be the source for wide variety of flavonoids. Moreover, these plant part specific compounds can be used as biomarkers for the identification of plant material or herbal drugs. Overall, the present study provides for the first time a comprehensive analysis of the phenolic components of this herb which may be helpful not only to understand their usage but also to contribute to quality control as well.

## 1. Introduction 

Bark decoction of *S. asoca* (Roxb.), De. Wilde (*Caesalpiniaceae*), has been mentioned as one of the most famous Indian treatise *Charaka Samhita *(100 A.D.) for the treatment of various types of gynaecological disorders. Bhavprakash Nighantu, another Indian treatise, referred to it as a uterine tonic for regularizing the menstrual disorders. Bark of the plant is well reported for its stimulating effect on endometrium and ovarian tissues and being used to treat menorrhagia. *S. asoca* contains significant amounts of phenolic compounds that are considered to be the biologically active components. Water extracts of the plant parts are being used to prepare various Ayurvedic and herbal drugs being rich source of catechin, epicatechin, epigallocathechin, and their polymers and glucosides [1, 2]. Catechins are well reported for various kinds of biological activities and are useful for the symptomatic treatment of several gastrointestinal, respiratory, and vascular diseases. The antioxidant activity of flavonoids has been studied with regard to retarding the aging of cells and protection against cancer and coronary or cardiovascular disease [3–5].

Various techniques are in use to identify phenolic compounds such as thin layer chromatography, high performance thin layer chromatography, gas chromatography, UV detection, high performance liquid chromatography (HPLC), and mass spectrometry. These methods are useful to detect a limited number of known compounds but are not applicable for the characterization of unknown polyphenols in crude mixtures. Quadrupole time-of-flight mass spectrometry (Q-TOFMS) is excellent technique to analyze multicomponents in the complex herbal extracts due to accurate mass measurement, high resolution, and ion separation [6]. Rapid data mining procedures and aligning algorithm tools have been used to process huge raw data generated from metabolome analyses [7]. These processed data were thereafter used successfully in various pharmacophysiological studies such as disease diagnostics, human nutritional science, and drug discovery [8, 9].

In the present study, HPLC coupled with Q-TOFMS in positive mode was used to generate nontargeted MS^*n*^ data from various crude extracts prepared by taking different parts of *S. asoca*. As on date scanty information is available from *S. asoca*, rather no one reported a comprehensive profile of phenolic compounds from this plant. Therefore, nontargeted MS^*n*^ data was generated and processed by using Mass Hunter qualitative software for identification of phenolic compounds from the various prepared extracts of *S. asoca*.

## 2. Experimental 

### 2.1. Reagents

Standard compounds and solvents lidocaine, D-camphor, 5-7-isoflavone, formic acid and acetic acid (HPLC grade), acetonitrile, and formic acid and water (LCMS grade) were purchased from Sigma-Aldrich (St. Louis, MO. USA). Phenolic standards protocatechuic acid, coumaric acid, and quercetin were obtained from Sigma (St. Louis, MO, USA). Epicatechin, catechin, gallic acid, ferulic acid, and caffeic acid were purchased from Fluka (Buchs, Switzerland). The purity of the standards was more than 98%, and stock solutions were prepared as at 1 mg/L in methanol. Working standard solutions were made by diluting the stock solutions with mobile phase of HPLC.

### 2.2. Plant Material

Bark, regenerated bark, leaves, and flowers of *S. asoca* were collected from Botanical Garden of National Research Institute of Basic Ayurvedic Sciences, CCRAS, (Department of AYUSH), Nehru Garden, Kothrud, Pune, in February 2012 (winter season). The collected plant materials were identified, and voucher specimens (no. 207) were kept at the medicinal plant museum of the institute.

### 2.3. Extraction and Sample Preparation

Fresh plant materials were extracted overnight (at 25 and 70°C) with deionized water (Direct-Q, Millipore) and methanol in sequence (1 : 1 w/v). Extraction steps were repeated three times to ensure complete recovery of metabolites. The pooled supernatant phases were filtered through 0.22 *μ* filters (HiMedia), concentrated under vacuum to dryness (FreeZone 4.5 Labconco, CA, USA), and stored at −80°C till further use. All the samples were given abbreviated name as: bark water, hot water, and methanol extract (B), regenerated bark water, hot water, and methanol extract (RB), leaves water and hot water extract (L), and flower water and hot water extract (F). The extracts were reconstituted in HPLC mobile phase (5.0 mg/mL) for further analytical studies. Standard compounds lidocaine, D-camphor, and 5-7-isoflavone (5.0 ppm) were mixed in the samples.

### 2.4. HPLC

Experiments were performed on Agilent 1290 Infinity Series HPLC interfaced with an Agilent 6538 Accurate-Mass Q-TOF. A ZORBAX 300SB reverse phase column (C18, 4.5 mm × 250 mm, and 5 *μ*m particle size) with guard column of same diameter and pore size was used at a flow rate of 0.2 mL/min. The column temperature was maintained at 40°C. The mobile phase used for HPLC was combination of solvent A (0.1% formic acid in water) and solvent B (0.1% formic acid in acetonitrile). The gradient was varied linearly 5–10% in 15 min, 10–45% in 22 min, 45–65% in 30 min, 65–90% in 35 min, and finally to 5% B at 45 min. Sample volume of 20 *μ*L was injected by autosampler.

### 2.5. Q-TOFMS Conditions

Q-TOFMS was calibrated and tuned as recommended by the manufacturer to get accuracy less than 5 ppm. Instrument was operated in positive ion polarity mode and extended dynamic range (1700 *m/z*, 2 GHz) with following parameters: gas temperature 350°C, nebulizer 50 Psi, gas flow 11 L/min, capillary voltage 3500 V, nozzle 500 V, skimmer voltage 65 V, octapole RF 250 V, octapole DC1 48 V, and fragmentor voltage 175 V. MS^*n*^ data was collected in total ion counting mode, and spectra were acquired in the range 100–1100 *m/z* with acquisition rate 3 spectra s^−1^. To assure the mass accuracy of recorded data, standards of lidocaine and 5, 7-isoflavone were infused with samples along with continuous internal calibration with the use of signals at a range of *m/z* 121.05 to *m/z* 922.0098 (as per instrument standards).

## 3. Results and Discussion

### 3.1. HPLC/MS/MS Conditions Optimization

The HPLC-Q-TOFMS was tested with several basic and acid ionizers, but formic acid 0.1% was found to be most suitable among the tested conditions to resolve most of the compounds present in the crude extracts. In this condition ionic strength became appropriate, and the signal-to-noise ratio increased in the positive ion mode. However, negative mode also gets refined, but positive mode showed better ionization; therefore, it was selected to study the extracts. Being the crude extracts, several gradient profiles were tested, but used gradient profile allowed maximum separation of compounds in the extracts. Mixed standard solutions were tested in order to establish the optimum MS^*n*^ conditions. The fragmentation voltage was varied from 50 to 250 V and the collision energy from 5 to 45 V. The best results were obtained at fragmentation voltage 175 and ramping collision energy.

### 3.2. Analysis Catechins from Standards and* S. asoca* ****Extracts

**Figure 1:**
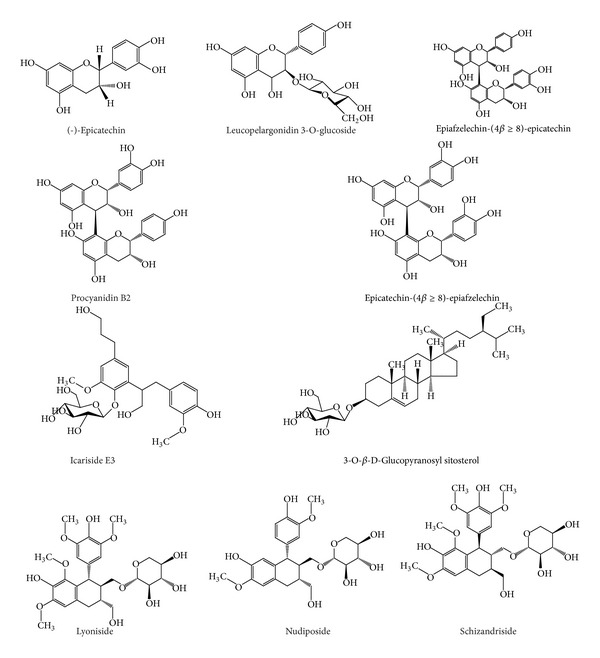
Structures of some known compounds from *S. asoca. *

**Figure 2:**
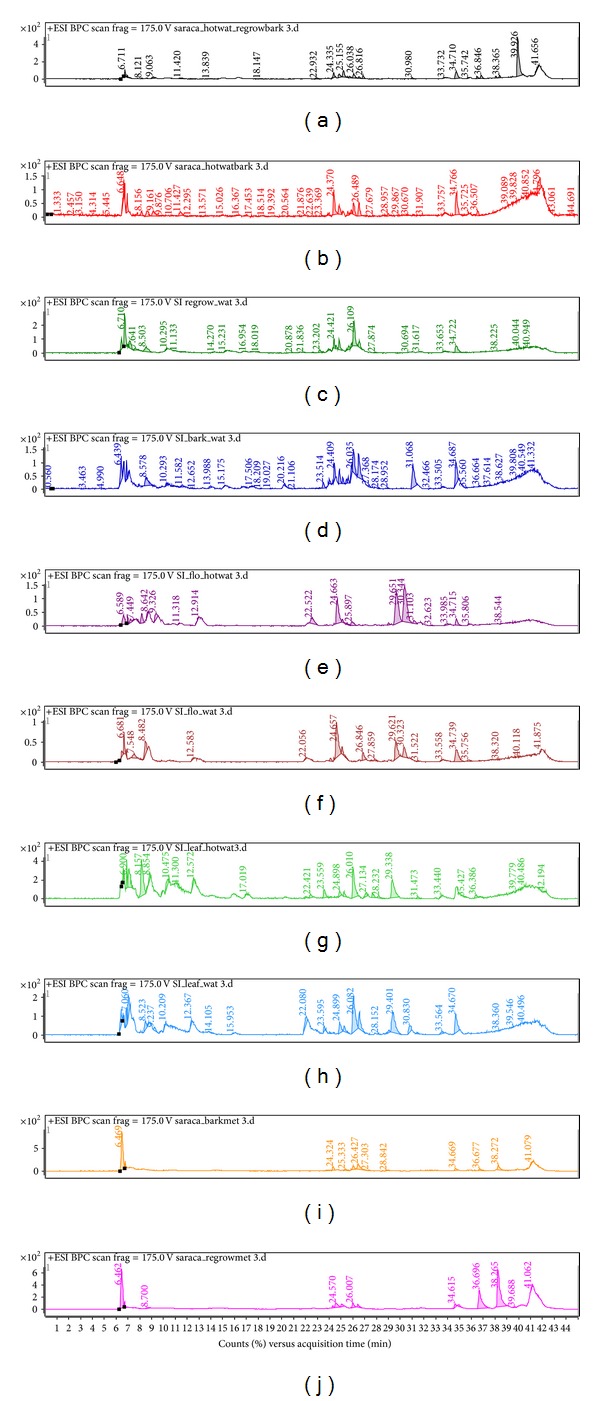
BPC scans of *S. asoca* regenerated bark hot water (a), bark hot water (b), regenerated bark water (c), bark water (d), flower hot water (e), flower water (f), leaves hot water (g), leaves water (h), methanol bark (i), and regenerated bark methanol (j) extracts. Peaks assignment is listed in Table 1.

Figure 1 is showing some of important and previously known compounds identified from *S. asoca*. Standard MS^*n*^ spectra of some important compounds were obtained under positive electron spray ionization (+ESI) conditions as discussed previously. The spectra generated for catechins by +ESI gave the protonated molecule [M + H]^+^ and some fragments even at relatively low fragmentation and collision energy voltages. Catechin, (−)-epicatechin, and (−)-epigallocatechin yielded the protonated molecule [M + H]^+^ (*m*/*z *291) along with other characteristic ions at *m/z* 123, 139, 161, and 207 [10]. For instance, other fragments of *m/z* 207, 219, and 275 were observed in the spectra. The retro-Diels-Alder fragmentation ions are reported as characteristic fingerprints for the presence of catechins in complex matrices. [M + H-galloyl + H–H_2_O]^+^ is a general fragmentation pattern observed for all catechin gallates and gallocatechin gallates [11]. Fragmentation of the predominant positive ions in nontargeted MS^*n*^ mode was used to obtain information about the molecular masses of conjugates and sugar moieties bound to the aglycones. The total ion chromatograms in positive mode of the extracts in Figure 2 are showing visual changes in profiles of different parts. The positive full-scan LC/MS analysis produced peaks for derivatives of catechins which were identified by scanning the characteristics fragment ions and matching standards available in the literature (Table 1).

C- and O-glycosides were identified on the basis of previous reports. In the positive ion full-scan mass spectrum, the C-glycosides showed only the prominent [M + H]^+^ ion with losses of 120 and 150 u (X + [M + H–120]^+^ and X + [M + H–150]^+^). The analysis of protonated C-glycosides by ESI-Q-TOFMS has proven that the ions of X + [M + H–90]^+^, X + [M + H–120]^+^, and X + [M + H–150]^+^ are the characteristic product ions for polyphenol C-glycosides, and the losses of 120 and 150 u are more favourable [12], whereas in polyphenol O-glycoside X+ [M+H–162]^+^ was characteristic ion due to neutral loss of 162 u in the product ion spectra.

Using the standards and identification of characteristic ions, 34 catechins and their derivatives were identified from the samples. The gradient of water containing 0.1% formic acid and acetonitrile 0.1% formic acid method produced well-shaped peaks for (−)-epicatechin, catechin, and epigallocatechin at 24.447, 25.261, and 23.8 min, respectively [10]. (−)-Epicatechin and catechin were differentiated on the basis of their retention time related to spectra of standard compounds. Moreover, several new derivatives of catechin were identified, and some remain unidentified (Table 1). Catechin-O-glucoside and catechin di-O-glucoside were identified for the first time as these give characteristic peaks of catechin along with neutral loss of 162 u due to loss of glucose moiety. Six catechin derivatives were found throughout the sample. Other catechin derivatives were observed to be specific with respect to plant parts which can be used as plant part specific markers and can be helpful in standardization of herbal drugs. Regenerating bark was found to have maximum number of catechin derivatives and tannins which might be induced under stress of regeneration and to prevent infections due to damage in bark.

**Figure 3:**
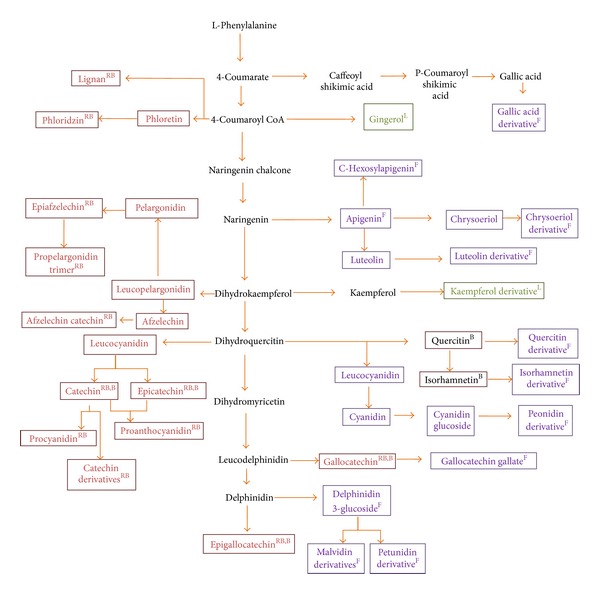
Plant part specific flavonoids biosynthesis pathway in *Saraca asoca*. Brown, violet, and green boxes correspond to the compound present in regenerated bark (RB), flower (F), and leaves (L) of *S. asoca*, respectively.

On the basis of inclusive analysis of phenolic compounds, pathway of flavonoids and their derivatives biosynthesis in *S. asoca *were explored (Figure 3). These compounds showed unique pattern of metabolites in the plant parts. In the study, *S. asoca* was found to be a rich source for catechins that accumulate in all the organs especially in bark. Contrary to this, epicatechin-3-O-gallate, and epigallocatechin-3-O-gallate were observed in the leaves and flowers of this herb.

### 3.3. Analysis of Flavonoids from Standards and *S. asoca* Extracts

Samples of *S. asoca* were analysed for flavonoids and found to have apigenin, kaempferol, peonidin, quercetin, isorhamnetin, chrysoeriol, and their derivatives. However maximum numbers of flavonoids were observed in the flower extracts of herb. In this study, total 34 flavonoids were characterized. Most of them were unambiguously identified by comparing retention times and MS data with those of the reference standards and discussed in the literature. Concerning the presence of aglycones in *S. asoca*, up to now several aglycones have been described in the literature [13]. The product ion spectra of apigenin, kaempferol, peonidin, quercetin, isorhamnetin, and chrysoeriol (Figure 1) were identified by comparing the product ion spectra and retention times with those of standards provided with a useful tool for the confirmation of the presence of these six aglycones in *S. asoca* extracts for the first time. Aglycones were identified by product ions generated by neutral losses of CH_3_ group, H_2_O, and CO as described previously [14, 15]. Glycosides of flavonoids were identified as described previously in case of catechins counting the loss of 162, 150, 120, and 90 u which are characteristics of flavonoids O- and C-glucosides. Total ion chromatogram was screened for loss of 162, 150, and 120 u. All the fragments were assigned with an accuracy of less than 5 ppm with few exceptions. Aglycones were fixed by comparing the product ions from standards and the literature. Resulted flavonoid glycosides are given in Table 2. Peonidin, quercetin, delphinidin, isorhamnetin, petunidin, and malvidin glycoside were mainly observed in flowers as shown in biosynthesis pathway (Table 2, Figure 3).

### 3.4. Analysis of Other Compounds from *S. asoca* Extracts

Compounds other than catechin and flavonoid derivatives were identified with help of standard mass spectral libraries from http://spectra.psc.riken.jp and http://www.massbank.jp [16, 17].  Table 3 is showing compounds and their product ions. Unidentified compounds were mentioned as unknown or derivative of known compounds.

## 4. Conclusions

The rational use of *S. asoca* plant parts for declining uterine diseases is mainly due to presence of flavonoidal glycosides, catechins, oligomeric procyanidins, and steroids. The detailed identification of the phenolic composition of *S. asoca* provides the background necessary to evaluate the biological activity of the identified compounds and to develop an understanding of the potential benefit of the herb. A number of steroidal compounds were also observed in all plant parts but could not be identified very well due to limited fragmentations. The qualitative and comparative method showed good results in terms of identification of flavonoids. Variety of catechin derivatives were found to be elevated in regenerating bark. One possible reason for the elevation of flavonoids could be the protective effect of these compounds against plant infections. Part specific compounds as shown in Tables 1, 2, and 3 can be used as biomarkers for the identification of plant material or herbal drugs. This comprehensive analysis of the phenolic components of herb will be helpful not only in the quality control of this herb and its products but also in understanding medicinal importance of different parts of the herb. Besides this, the content of desire compound can be enhanced in specific part of the plant by using metabolic engineering where the present data will be very useful and supportive.

## Figures and Tables

**Table 1:** Identified catechins and their derivatives in different parts of *S. asoca*.

S. no.	RT	Name of compound	Product ions (*m/z*)	Calculated mass	Exact mass	Sample*
1	6.23	Gallocatechin 3-O-gallate	139.02, 289.2, 361.341, and 459.59	458.137	458.08	F
2	7.03	Gallic acid hexoside	111.001, 159.234, 171.0423, 219.161, and 239.128	332.073	332.071	F, B, RB
3	7.30	Gallic acid derivative	153.249, 171.034	193.0352		ALL
4	7.72	Protocatechuic acid	109.04, 127.062	155.051	154.031	F, L
5	8.20	Catechol	111.006	110.006	110.036	ALL
6	10.90	Gallic acid	109.024, 127.131, and 153.323	170.041	170.021	F, L
7	21.99	Catechin derivative	127.021, 139.0156, 165.012, 271.213, 291.213, 409.210, and 569.351	740.221	—	RB
8	22.41	Catechin derivative	139.237	351.825	—	L
9	23.64	(epi)Catechin-(epi)catechin-(epi)catechin	127.138, 151.335, 163.411, 245.710, 301.779, 409.722, 427.707, 451.665, 527.526, 578.477, and 715.239	866.081	866.211	RB, B
10	23.70	Procyanidin B3	127.131, 275.741, 287.749, 291.742, 409.709, 417.690, and 427.690	578.516	578.14	B
11	23.80	Procyanidin B2	127.13, 139.23, 289.163, 291.177, and 409.206	578.463	578.53	
12	23.83	Catechin diglucoside	123.102, 139.023, 165.041, 285.101, 291.179, 315.179, 383.277, and 453.202	598.294	—	RB
13	23.88	Tannin	127.103, 139.236, 163.23, 287.231, 301.268, 393.213, and 409.321	724.25	724.242	RB
14	23.95	Procyanidin B1	127.131, 139.237, 163.405, 271.743, 287.749, 291.781, 301.766, 409.708, 427.686, and 543.509	578.463	578.14	ALL
15	24.18	Procyanidin C1	127.137, 139.244, 289.775, 291.756, 409.722, 545.504, and 577.476	866.082	866.205	RB, B
16	24.37	Catechin derivative	127.013, 139.137, 289.265, 301.215, 393.243, 409.213, 427.209, 464.204, and 563.231	871.099	—	RB
17	24.80	Epiafzelechin	107, 139.236, 149.33, 169.44, 191.57, 233.67, and 257.74	274.779	274.084	B, RB
18	24.83	Hydroxy catechin	123.103, 139.243, 151.355, 163.412, 181.520, 207.637, and 215.668	302.798	—	B
19	24.87	Catechin glucoside rhamnose	139.037, 275.279	583.507	—	B
20	24.97	Epicatechin	123.097, 139.098, 147.104, 165.132, and 207.229	291.237	290.27	ALL
21	25.05	Galloyl-isorhamnetin	317.213	468.205	—	F, L
22	25.29	Catechin	123.097, 139.098, 147.104, 165.132, and 207.229	291.215	290.27	ALL
23	25.30	Afzelechin-(4alpha→8)-catechin	107.051, 139.245, 147.311, 231.702, 273.772, 287.761, 291.754, 393.751, 409.722, 411.7024, and 427.705	562.574	562.15	B, RB
24	25.48	Proanthocyanidin trimer	127.0124, 139.123, 151.133, 163.133, 247.243, 271.254, 287.261, 301.279, 397.232, 409.272, 427.2046, 449.256, 534.133, 577.173, 679.280, 695.2561, and 713.238	864.036		B
25	25.53	Dicatechin gallate	287.248, 409.203, and 579.263	730.193	—	B
26	26.04	Tricatechin gallate	239.02, 247.134, 279.265, 518.135, and 579.238	1018.944	—	F
27	26.22	Propelargonidin trimer	119.009, 139.123, 151.023, 231.168, 289.262, 300.277, 325.275, 329.255, 381.242, 393.326, 409.204, 419.17, 425.175, 435.187, 451.148, 471.166, 546.119, 555.199, 577.144, 680.1736, and 699.246	850.055	850.21	RB
28	26.44	Catechin-(4alpha→8)-gallocatechin-(4alpha→8)-catechin	139.012, 153.021, 271.243, 287.246, 331.231, 417.201, 544.141, 563.153, 587.127, and 714.2103	881.99	882.22	F
29	26.76	(−)-Epicatechin-3-O-gallate	123.09, 139.237, 153.321, 165.433, 273.760, and 291.781	442.661	442.09	F
30	27.40	Catechin O-glucoside	123.102, 139.023, 165.041, 291.179, 367.234, and 411.259	452.202	—	RB
31	27.80	Lignan	137.012, 145.123, 151.156, 167.125, 181.174, 189.126, 285.145, 317.174, 361.267, and 465.276	464.136	—	RB
32	28.32	Petunidin gallate	317.782	485.693	—	L
33	29.80	(−)-Gallocatechin	139.243, 289.345, and 291.786	306.004	306.07	RB
34	37.55	Cyanidin 3-(2G-galloylrutinoside)	748.492	747.49	747.482	RB

*Abbreviations B, F, L, and RB in sample column represent bark, flower, leaves, and regenerated bark, respectively.

**Table 2:** Identified polyphenols and their glycosides in different parts of *S. asoca*.

S. no.	RT	Name of compound	Product ions (*m/z*)	Calculated mass	Exact mass	Sample*
1	11.96	Kaempferol	112.01, 147.03, 163.134, 211.224, and 243.232	286.264	286.240	L
2	15.18	Kaempferol 3-diglucoside-7-glucoside-p-coumaloyl	471.167	918.198^#^	918.191	B, F, RB
3	15.43	C-Hexosyl-apigenin	283.125, 367.723	528.528	528.520	F
4	21.40	Quercetin-3-rhamnoside	129.01, 141.025, 233.177, 287.147, 303.281, and 449.214	448.218	—	F, L
5	22.10	Petunidin-3-O-beta-glucopyranoside	317.125	479.122^#^	479.118	F
6	22.33	Unknown gingerol type glycoside	139.123, 181.143, 265.213, 33.242, and 351.273	512.251^#^	—	L
7	22.59	Pentahydroxyflavone-O-glucoside	129.12, 137.153, 153.123, and 305.833	466.265^#^	466.157	F, L
8	22.72	C-Hexosyl-luteolin O-hexoside, O-pentoside	299.213, 329.142, 353.125, and 383.217	743.286	742.278	FW
9	23.20	Peonidin-3-O-*β*-galactopyranoside	301.145	463.184^#^	463.124	F, L
10	23.57	Dihexosyl quercetin	303.124, 465.213	626.134	626.150	F
11	23.78	Quercetin	123.10, 137.24, 151.33, and 285.77	302.796	302.04265	B, RB
12	24.10	Quercetin-3′,7-di-O-glucoside	287.249, 449.364	610.411	610.52	F
13	24.14	Isorhamnetin sophorose	317.263, 479.223	640.167^#^	640.160	F
14	24.52	6-Hydroxykaempferol	123.102, 139.214, 147.31, 151.3358, 165.43, 181.518, 193.57, 207.63, 215.668, 243.722, 261.67, and 285.785	302.796	302.042	B
15	24.52	C-Hexosyl-chrysoeriol O-hexoside	301.124, 463.256	624.204^#^	624.17	F
16	24.70	3,5,7,2′,6′-Pentahydroxyflavone	215.727, 243.82, and 289.811	306.864^#^	304.058	L
17	24.77	Malvidin-3-galactoside	331.772	493.112^#^	493.134	F
18	24.80	Peonidin glucoside derivative	301.767, 463.654	776.145^#^	—	F
19	24.88	Peonidin-3,5-O-di-*β*-glucopyranoside	286.0235, 301.0235	625.241^#^	625.176	F
20	24.90	Phloridzin^#^	275.421	437.542^#^	436.136	RB
21	24.99	Peonidin-3-O-alpha-arabinopyranoside	133.023, 177.253, 301.271, and 415.123	433.105^#^	433.113	F
22	25.03	(+)-Dihydrokaempferol	107.04, 123.09, 127.13, 139.23, 149.29, 163.407, 166.472, 179.434, 215.654, 243.715, 259.759, 271.744, and 289.763	288.763	288.063	ALL
23	25.08	3-O-Hexosyl-quercetin	133.197, 145.295, 153.356, and 301.178	464.075	464.10	F
24	25.22	Leucopelargonidin 3-O-glucoside	137.124, 291.178, and 303.155	452.185	452.131	ALL
25	25.26	Apigenin	107.043, 119.003, 149.013, 153.123, 174.155, 215.165, 228.213, and 243.214	270.103	270.05	ALL
26	25.43	Malvidin-diglucoside	331.275, 493.213	655.257^#^	655.187	F, L
27	25.46	Isorhamnetin-3-coumaroylglucopyranoside	317.275, 463.266	624.224^#^	—	B
28	25.70	Isorhamnetin	115.043, 123.176, 147.109, 165.133, 257.177, 297.020, and 302.054	316.02^#^	316.06	B
29	26.5	Delphinidin-3-O-*β*-glucopyranoside	303.213	465.231^#^	465.103	F
30	27.28	Quercetin-3-O-Arabinoside	131.01, 137.024, 151.125, 181.123, 257.263, 285.214, 303.214, and 360.225	434.2	434.214	B
31	27.43	Quercetin 3,4′-di-glucoside-3′-(6-caffeoylglucoside)	625, 787, and 487.685	950.265	950.257	RB
32	30.20	7-Acetyloxy-2-methylisoflavone	107.08, 111.077, 121.134, 125.068, 151.100, 161.187, 179.194, 193.170, 221.191, 237.146, 249.178, 259.176, and 277.103	294.102^#^	294.089	F
33	30.47	Peonidin	286.0235, 301.068	301.067^#^	301.071	ALL
34	34.60	Isorhamnetin-3-O-glucoside	302.155, 317.013	478.032^#^	478.111	F

*Abbreviations B, F, L and RB in sample column represent bark, flower, leaves, and regenerated bark, respectively.

^#^Compound detected for the first time in *S. asoca*.

**Table 3:** Other compounds identified in different parts of *S. asoca*.

S. no.	RT	Name of compound	Product ions (*m/z*)	Calculated mass	Exact mass	Sample*
1	7.10	L-Homocitrulline	100.123, 127.061, 155.280, and 173.213	—	189.111	ALL
2	10.22	Dehydrogenated-decarboxy-neobetanin	341.771	502.598	—	RB
3	15.70	Ecdysone	123.045, 233.213, 279.253, 297.256, 313.257, 325.252, 393.383, 429.266, 447.256, 465.225, and 482.167	482.167 (M + H + NH_3_)^+^	464.122	L
4	20.26	17-Decarboxy-betanin	345.289	506.217	506.152	B, RB
5	22.02	Triterpenoid hexose	126.98, 323.711, 429.738, and 505.594	666.330	666.40	F, L
6	22.35	11-Hydroxy-sec-O-*β*-D-glucosylhamaudol	293.794	454.695	—	B
7	24.3	D-(+)-Cellotriose	203.201, 325.298, 343.231, and 487.241	504.208	504.169	B, RB
8	24.65	Unknown diglucoside	323.245, 485.6	647.343	646.335	LHW
9	24.94	14-Hydroxycarpesterol	127.012, 139.123, 163.102, 257.251, 275.231, 291.215, 301.253, 337.296, 401.196, 409.203, 427.196, and 560.293	578.222	578.22	ALL
10	25.44	Icariside E3	115.023, 145.125, and 188.156	524.206	524.225	F, L
11	25.50	3-O-beta-D-Glucopyranosyl sitosterol^#^	397.213, 415.282	576.406	576.438	B, RB
12	26.11	7-Dehydrocholesterol glucoside	120.8, 133.1, 159.2, 247.2, 259.2, 368.2, and 385.2	546.2	—	RB, B
13	26.50	Phytolaccagenic acid 3-O-glucose (1′′→3′) galactose	249.772, 517, and 679	840.329	840.321	RB
14	27.90	Unknown	123.09, 153.121, 271.25, 394.243, and 542.197	882.993	—	F
15	28.2	Tyramine-betaxanthin	163.149, 249.244, and 287.219	330.244	330.12	B, RB
16	34.36	4-Methylthio-n-butyl glucosinolate	186.001, 286.23, 316.993, and 398.505	477.900	477.984	B, RB
17	37.90	Tripalmitin type compound	393.89, 313.9816, 239.89, 155.333, and 137.3193	554.672	—	F

*Abbreviations B, F, L, and RB in sample column represent bark, flower, leaves, and regenerated bark, respectively.
